# Systematic literature review on biopsychosocial factors in the surgical waiting list

**DOI:** 10.1016/j.clinsp.2026.100906

**Published:** 2026-03-27

**Authors:** Fabián Silva-Aravena, Jenny Morales, Leonidas López Canales

**Affiliations:** Facultad de Ciencias Sociales y Económicas, Universidad Católica del Maule, Avenida San Miguel 3605,Talca 3460000, Chile

**Keywords:** Surgical waiting lists, Biopsychosocial factors, Patient prioritization, Elective surgery, Health-related quality of life (HRQoL)

## Abstract

Prioritizing patients on elective surgical waiting lists is one of the strategies to reduce waiting times for care, improve Health-Related Quality of Life (HRQoL), and optimize clinical resources. According to the World Health Organization (WHO) recommendations, including biopsychosocial factors in the Clinical Priority Assessment Criteria (CPAC) is critical for an equitable and efficient allocation of surgical treatments. Long waiting lists, decreased Health-Related Quality of life (HRQoL), lack of capacity of health care facilities, and lack of policies to improve these problems negatively affect the population worldwide. This article presents a systematic literature review to identify biopsychosocial factors that can be used for prioritizing patients on surgical waiting lists. 71 articles were studied, finding that the most frequently mentioned biopsychosocial factors are pain and/or discomfort, followed by patient age, disease severity, comorbidities, expected complications after the procedure, ability to work and/or study, efficacy of the procedure and/or expected improvement, time on the waiting list, ability to participate in family/social activities, impact on the disease if surgery is delayed, need for a caregiver, effect on the procedure if surgery is delayed, composition and size of the surgical team, being a caregiver, length of hospital stay, urgency of the procedure, among others. These findings reinforce the importance of a comprehensive assessment of the patient and the environment to improve clinical outcomes and equity in access to surgical interventions by integrating the patient and physician perspectives in pursuit of better outcomes.

## Highlights

• Review of biopsychosocial factors in Surgical Waiting List prioritization (SWL).
• Identification of frequent biopsychosocial factors in managing patients on SWL.
• Association between biopsychosocial factors by clinical area in patients on SWL.
• A holistic approach that balances both patient and physician perspectives.

## Introduction

Surgical waiting lists are a significant political and economic concern in health systems.^1^ They are a key performance indicator, influencing resource allocation and policy effectiveness. The implications of backlogs on waiting lists are a growing concern^2^; they cause patient dissatisfaction due to anxiety, frustration, discomfort, uncertainty, and anger.^3^^,^^4^ Furthermore, once the patient’s symptoms worsen, the quality of life is compromised, absences from study and/or work increase, and it can even cause the patient’s death.^4^ According to the World Health Organization, reducing waiting times and delays is a key element of quality in health services^5^ and in turn, the Sustainable Development Goals (SDGs) underline that quality of health is a crucial element of universal health coverage (see SDG target 3.8). Every year, between 5.7 and 8.4 million deaths in low- and middle-income countries are attributed to poor-quality care, which represents up to 15 % of deaths in those countries.^5^

Health systems around the world are collapsing due to ever-increasing demands for care,^6^ and to this are the limited resources available to care for patients.^7^ However, much evidence exists that increasing resources alone is not the solution, as the average waiting time decreases, but the queue gets longer.^8^ Added to this are the difficulties faced by general surgery residents, including lack of experience, work-related challenges, and personal concerns,^9^ they also affect the increase in waiting lists. When the waiting time to receive the required medical care exceeds the established limits, waiting lists increase the system’s costs, increase the risk of complications, prolong or worsen the illness, and delay the patient’s recovery.^10^

Several studies have explored surgical waiting lists’ challenges and potential solutions^11^ and^12^ focused on patient prioritization methods to reduce waiting times for elective surgery.^11^ stressed the need for explicit prioritization tools with standardized scoring systems while^12^ highlighted the ongoing development of priority scoring tools in several countries. These studies collectively underline the need for effective strategies to address the challenges of surgical waiting lists, both from the perspective of patients and professionals.

### Impact of Covid

The Coronavirus (COVID-19) pandemic has significantly impacted healthcare systems and academic programs for surgical residency training,^13^, ^14^, ^15^ the diagnosis, referral, and treatment of diseases,^16^ the reduction of elective or low-priority surgical interventions, outpatient visits^17^^,^^18^ and the postponement, delay or pause of elective surgeries such as urological surgeries,^19^ pediatric orthopedics,^18^ cardiac, aortic and vascular surgery,^20^^,^^21^ bariatric,^22^ oncological^23^ among others.^24^ Added to the above is the regulatory limitation of “elective surgeries” that negatively impacted patients, running perioperative and postoperative risks^22^^,^^25^ therefore, appropriate prioritization policies for patient prioritization systems for surgical waiting lists and COVID-protected pathways^16^ and future pandemics are vital in helping mitigate the negative effects.

### Biopsicosocial

The term “biopsychosocial” was first coined by Roy Grinker in 1952 and later expanded by George L. Engel in 1977.^26^ This model emphasizes the interconnectedness of biological, psychological, and social factors in understanding and treating mental disorders. Healthcare systems designed around biomedical acute care models struggle to improve patient-reported outcomes and reduce healthcare costs. Consequently, there is a greater need to apply the biopsychological model to healthcare management.^27^

The biopsychosocial model is widely used in research into complex health interventions, is the basis of the World Health Organization’s International Classification of Functioning (WHO ICF), is used clinically, and is used to structure clinical guidelines.^27^ A biopsychosocial approach to surgical waiting lists is crucial to prioritize patients and understand the impact of delays on their well-being.^28^^,^^29^ developed a decision support system that considers biopsychosocial criteria, allowing for improved prioritization and reduced waiting times. This approach is aligned with the recommendation of^30^ to assess psychosocial factors in surgical evaluation to prevent conflicts and adverse outcomes^31^ also highlights the need to consider surgical delay’s physical, psychological, and social consequences, stressing the importance of a holistic approach to surgical waiting lists.

In the world, strategies have been oriented in at least two directions: defining waiting times and forcing providers to comply with said guaranteed times, similar to the Chilean General Regime of Explicit Guarantees in Health (GES, from its acronym in Spanish). However, this causes GES diseases to be prioritized and non-GES diseases that require surgical intervention to be left aside, causing an increase in the non-GES waiting list.^32^

According to the national plan for non-GES waiting times in Chile, in December 2017, a proposal was received from the Health Planning Division of the Undersecretary of Public Health, which contains the prioritization strategy and waiting times, which includes an algorithm for prioritizing the resolution of non-GES waiting patients built based on 8 criteria (clinical diagnosis, age, and sex, associated chronic condition, number of previous hospitalizations, number of referrals on non-GES waiting list, presence of a GES pathology, use of medications and observed waiting time).^32^ However, other prioritization criteria remain to be evaluated, such as social factors (living alone, having dependents), quality of life, and behavior.

In Chile, the implementation of patient prioritization systems for surgical waiting lists using biopsychosocial factors is relatively new,^29^ implemented a decision support system was implemented for non-GES surgical waiting lists in a clinical otorhinolaryngology unit based on biopsychosocial criteria in a highly complex hospital in Chile. As a result, they managed to reduce the waiting list from 462 days to 282 days.

The literature presented shows that the problems associated with waiting lists can be addressed differently. They are essential and depend mainly on the patient’s specificity and environment. This SLR (Systematic Literature Review) seeks to identify the different biopsychosocial factors that affect the prioritization of surgical waiting lists to show the need to identify the most significant factors and in which clinical areas. For this purpose, the (PICOC) technique will be used, which is intended to update the knowledge and availability of these factors that will be the basis for creating new systems for prioritizing surgical waiting lists and developing surgical waiting list systems.

### Goals

This research aims to identify biopsychosocial factors in surgical waiting list prioritization. In the development of this work, we focus on:

• Identify the biopsychosocial factors frequently considered for managing surgical waiting lists.
• Identify psychosocial factors by clinical area in managing patients on surgical waiting lists.
• To explore the association between biopsychosocial factors by clinical area in patients on surgical waiting lists.

The article is organized as follows: Section 2 presents the research questions, the PICOC methodological design applied, and the details for data extraction, as well as the inclusion and exclusion criteria. Section 3 shows the analysis of the information found. Section 4 offers a discussion of the results obtained and their limitations. Finally, in section 5 authors present conclusions and associated future work.

## Methodology

A systematic review of the literature includes 3 phases, as explained^33^ and used^34^: (i) Planning the review, (ii) Conducting the review, and (iii) Reviewing the report, which presents the review results. Following the previous phases, we defined the review protocol for this systematic literature review as follows: data source, search strategy, selection criteria, data extraction, and data synthesis.

### Research questions

**Fig. 1:**
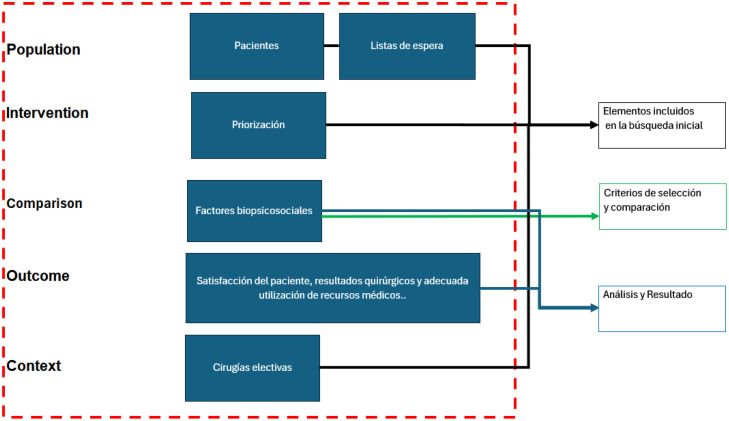
Conceptualization of the elements PICOC.

The research questions have been formulated using the PICOC (Population, Intervention, Comparison, Outcome, Context) method to guide the systematic review, which is generally used in systematic reviews in the fields of health and social services management, among others.^35^, ^36^, ^37^ The following PICOC criteria were defined for this research (Fig. 1, Table 1).

• Population (P): Patients on surgical waiting lists.
• Intervention (I): Criteria, factors, or forms of selection of patients on the surgical waiting list, that consider biopsychosocial dimensions.
• Comparison (C): Different surgical waiting list management approaches, excluding those considering biopsychosocial factors.
• Outcome (O): Patient satisfaction, surgical results, and adequate use of medical resources.
• Context (C): Elective surgeries of patients in clinical areas.

**Table 1:** Keywords and synonyms for prioritization, waiting lists, elective surgery, and patients.

Population / Keywords	Synonyms	Terms included in the search
Prioritization	health priorities - triage - prioritizing - prioritization preemption - preference - decision making	(priorit* OR triage OR “health priorities” OR preference)
Waiting lists	waiting list - queue - waiting times - waiting list - delay - priority	(“waiting time*” OR “wait time*” OR “waiting list*” OR “wait list*”)
Elective surgery	elective surgery - surgical elective - elective surgical procedure - non-urgent surgery - non-urgent unscheduled surgery	(“elective surgery” OR “surgical elective” OR “non-urgent” OR “unscheduled surg*”)
Patient	client - patient - service user	(patient*)

As mentioned above, we oriented the systematic review of the literature to the PICOC model. In Table 2, we present the three research questions defined for our work.

**Table 2:** Research questions.

ID	Research Question
RQ1	What are the cross-cutting biopsychosocial factors in prioritizing the surgical waiting list?
RQ2	What biopsychosocial factors are considered for prioritizing the surgical waiting list by clinical area?
RQ3	What are the biopsychosocial factors relevant to the clinical area?

### Data source

The following electronic databases were selected for the search for scientific articles: Scopus, WoS, and PubMed. These databases contain scientific articles oriented to health and the prioritization of patients on surgical waiting lists, which is the focus of this systematic literature review.

### Search strategy

The scientific articles to be collected must contain information related to the questions defined in Table 2. For this purpose, 3 search strings were developed, detailed in Table 3, example for Scopus.

**Table 3:** Search strings.

N°	Search strings
1	patient AND ("waiting time" OR "wait time" OR "waiting list" OR "wait list") AND (priorit* OR triage OR health priorities OR preference AND elective surgery OR surgical elective OR non-urgent OR unscheduled surg*)
2	(patient* OR "waiting time*" OR "wait time*" OR "waiting list*" OR "wait list*" OR "prioritization framework" OR "waiting time prioritisation" OR "priority system" OR "decisión support system" OR "patient prioritization system" OR "multi-criteria decision-making") AND (priorit* OR triage OR "health priorities" OR preference) AND ("elective surgery" OR "surgical elective" OR "non-urgent" OR "unscheduled surg*") AND (ability OR "Capacity to work" OR "capacity to study" OR "Need for a caregiver" OR "Being a caretaker" OR "Capacity to participate in family" OR "social activities" OR attitude OR interest OR psychological OR social OR biopsychology OR biopsychological OR biopsychosocial
3	prioritization AND (patient* OR clients OR client) AND (elective OR "elective surgical" OR procedure OR surgical OR waiting OR list OR biopsychosocial)

#### Inclusion and exclusion criteria

We defined the inclusion and exclusion criteria to address the search and selection of articles. The selection of articles was carried out according to the following criteria:

• Journal articles and conference papers.
• That investigated interventions and strategies aimed at reducing waiting time for surgery were included; this criterion was used by^11^ in their systematic review.
• Articles were included that addressed at least 2 of the 3 components of the term biopsychosocial (biological, psychological and social) that affect the patient, the clinical and hospital environment of elective surgical waiting lists.
• Articles that they search for, identify, or use factors for prioritizing patients on the elective surgical waiting list.
• Articles from 2015 to August 2024 were included.
• Articles that are in Spanish and/or English.

We exclude those articles that:

• Articles limited to biomedical criteria.
• Criterio 2: Articles that correspond to emergency surgery and do not contemplate elective surgery.

#### Selection and screening process

Duplicate articles were removed; we used the software command “Search for duplicates” in the “title” and “DOI” fields of the references. Then, we independently examined all references identified in the search and manually removed the remaining duplicates.

In the screening process, titles and abstracts were first analyzed to extract relevant articles. Whenever a discrepancy arose about the relevance of an article, the authors discussed the article until a consensus was reached.

Finally, the full texts of all extracted articles were read to select relevant ones according to our eligibility criteria.

#### Data extraction

The relevant information extracted from all the selected articles is as follows:

• Authors and year of the study.
• The journal and type of scientific article (poster, journal article, or conference).
• Biopsychosocial factors are identified in the article.
• Article contribution to the topic under study.
• The main results.
• Country where the study was conducted.
• Instrument used to identify biopsychosocial factors.
• Clinical area in which the study was conducted.
• Number and characterization of participants in the study.
• The type of health system in which the study was applied (public or private).
• Gender of the participants.
• The main conclusions of the study.
• The future works established in the study.

For articles related to the prioritization of patients on the elective surgical waiting list and/or those that develop a decision support system, the following specific information was also shown:

• Biopsychosocial factors identified in the decision support models identified by the authors of the scientific articles.
• Variables used in the decision support system.

#### Data synthesis

We used qualitative analysis, pooling and summarizing the results of the studies, and quantitative analysis was performed to describe the systematic review results. All included studies were analyzed using the same synthesis method, and the results were presented together and by relevant clinical areas. We extracted qualitative data related to the review objectives from all included manuscripts. In the extraction grid, we identified all biopsychosocial factors (e.g., pain, patient age, disease severity, time, and comorbidities, among others). Then, we assigned the clinical area in which these factors were applied. The quantitative data presented in our review are the numbers of occurrences of the extracted qualitative data in the included studies.

Network analysis is then applied to explore the scope and interactions of biopsychosocial factors and clinical areas, allowing us to analyze how these elements are integrated into a network of relevant elements. Within a red analysis, each predictor is represented by a node whose size indicates its relative frequency of appearance. The network (graph) that is the result of the analysis illustrates which predictors are closely related since these predictors are shown spatially close. Consequently, the network analysis reveals which predictors are the most essential inputs to the network. In this research, the analysis was performed using the UCINET software (version 6.625) used in other work.^38^

## Results

**Fig. 2:**
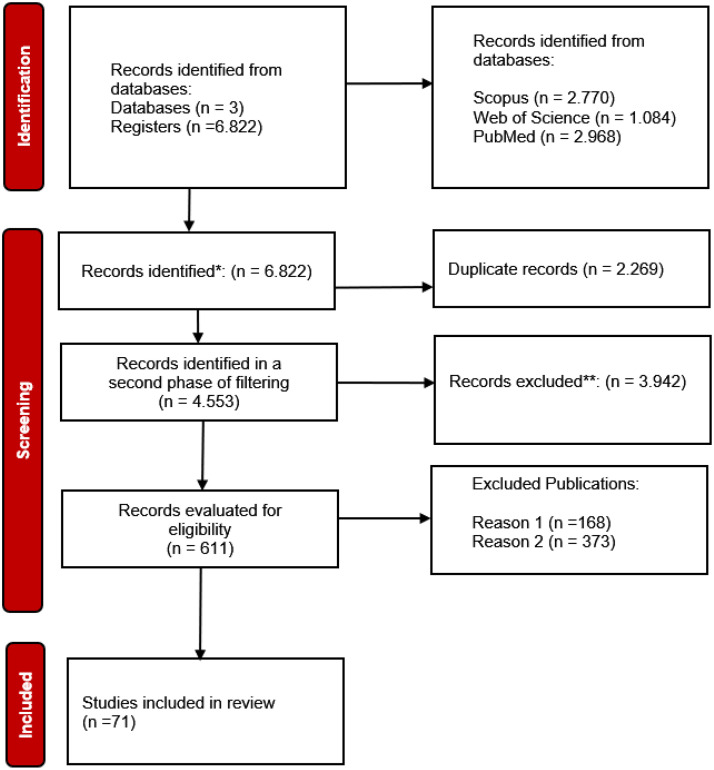
Flow Diagram according to PRISMA guidelines.

This work includes scientific articles found in Scopus, WOS, and PubMed; the results obtained are shown in Fig. 2. Our review highlights >240 factors, which we group into biological, social, and psychological factors.

The database search was conducted from July 10, 2024, to August 25, 2024, and was updated on September 1, 2024. After removing duplicates, 4683 scientific articles were examined. The titles and abstracts of 2963 articles were evaluated, leaving 302 full-text articles for analysis to determine their eligibility. Of these, 231 were excluded based on at least one exclusion criterion. Fig. 2 presents the flow chart for including the 71 relevant articles (see Appendix in section 6). The included articles were published between 2015 and 2024. Most studies were conducted in Canada, Spain, and New Zealand. The stated aims of these studies were mainly related to identifying biopsychosocial factors that may assist in prioritization criteria and creating prioritization tools.

This work includes scientific articles found in Scopus, WOS and PubMed, the results obtained can be seen in Fig. 2. Our review highlights >240 factors, which we group into three components: biological, social, and psychological factors.

**Fig. 3:**
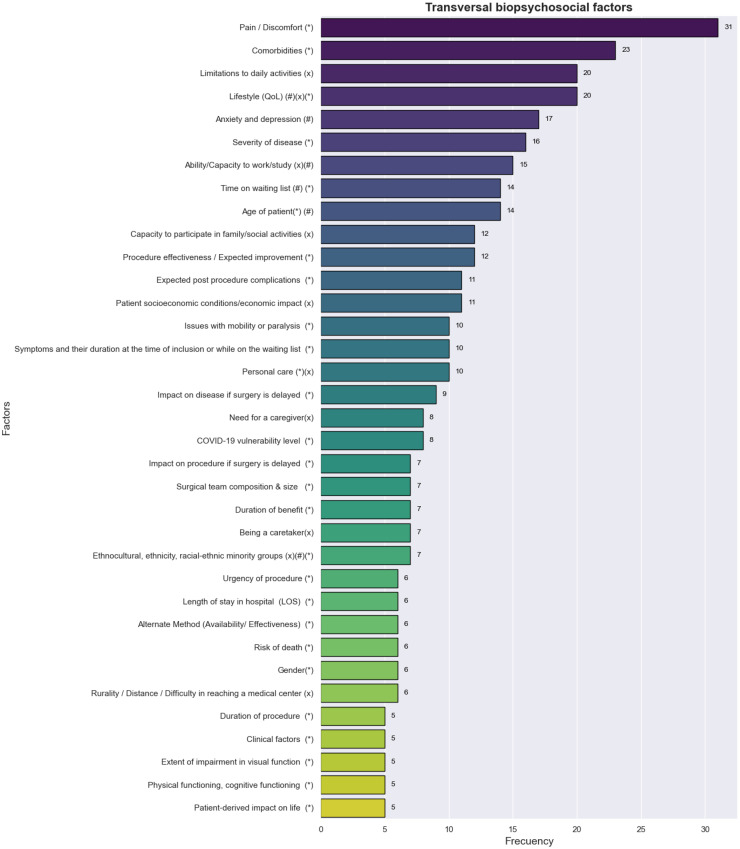
Transversal biopsychosocial factors: (*) correspond to factors whose component is biological, (#) corresponds to factors whose component is psychological, and (x) corresponds to factors whose component is social within the domains of (1) clinical care, (2) epidemiology, or (3) health services planning and financing.^43^ Comorbidities were considered by.^29^^,^^39^^,^^40^^,^^44^, ^45^, ^46^, ^47^, ^48^, ^49^, ^50^, ^51^, ^52^, ^53^, ^54^, ^55^, ^56^, ^57^, ^58^, ^59^, ^60^, ^61^, ^62^, ^63^.

The transversal biopsychosocial factors found in our systematic review can be seen in Fig. 3. Pain is the most frequent factor in the literature, found in clinical areas such as breast surgery, cholecystectomy, colectomy, diagnostic laparoscopy, hernia, pancreatic resection, perianal, stoma reversal, skin lesion excision, and thyroidectomy,^39^ orthopedics,^40^, ^41^, ^42^ ophthalmology, among others.

Below, we explain each of the biopsychosocial factors found in this research. For the purposes of this study, a value equal to 1 is assigned if the factor is mentioned in the article and 0 otherwise. The sum of these values represents the frequency of the factor, and to be considered transversal, it must be equal to or greater than 5 for graphic purposes.

1 **Pain** According to the medical definition established by the International Association for the Study of Pain (IASP), it is an unpleasant sensory and emotional experience related to an existing or potential tissue injury of each individual. For the clinical variable of pain, some authors, such as,^7^^,^^29^^,^^39^, ^40^, ^41^, ^42^, ^44^, ^45^, ^46^, ^47^, ^48^, ^49^, ^50^, ^51^, ^52^, ^53^, ^54^, ^64^, ^65^, ^66^, ^67^, ^68^, ^69^, ^70^, ^71^ demonstrated the relevance of this variable when prioritizing patients on the waiting list. In our study, the pain variable takes on a transversal character because it was considered in the area of ophthalmology by,^45^^,^^55^, ^71^, ^72^ in the area of otorhinolaryngology by,^7^^,^^29^ in the orthopedic area for^41^^,^^42^^,^^50^, ^53^, ^56^, ^57^, ^65^, ^73^, ^74^ concerning knee and hip,^52^^,^^54^ concerning the column, while other authors^39^, ^46^, ^47^, ^48^, ^51^, ^64^, ^67^ they considered it widely for multiple clinical areas.
2 **Comorbidities** are associated with worse health outcomes, more complex clinical management, and higher healthcare costs. The definition of comorbidity lies in its ability to explain a particular phenomenon of interest within the domains of (1) Clinical care, (2) Epidemiology, or (3) Health services planning and financing.^43^ Comorbidities were considered by.^29^^,^^39^^,^^40^^,^^44^, ^45^, ^46^, ^47^, ^48^, ^49^, ^50^, ^51^, ^52^, ^53^, ^54^, ^55^, ^56^, ^57^, ^58^, ^59^, ^60^, ^61^, ^62^, ^63^
3 **Limitation to daily activities**, it is a factor that is related to the difficulties in carrying out daily habits by the patient, before and after the operation, it is a direct component of the Quality of Life (QoL) measure by EuroQol-5D y EQ-5D- 3 L, this factor is considered by.^42^^,^^51^, ^52^, ^55^, ^56^, ^57^, ^64^, ^65^, ^66^, ^67^, ^68^, ^69^, ^72^, ^73^, ^75^, ^76^, ^77^, ^78^, ^79^, ^80^
4 **Lifestyle (QoL)**, the World Health Organization defines it as “an individual’s perception of their position in life in the context of the culture and value systems in which they live and in relation to their goals, expectations, standards and concerns”. In the literature, the patient’s quality of life is addressed by different dimensions (mobility, self-care, usual activities, pain and anxiety, and depression). This holistic factor is addressed by.^45^, ^51^, ^52^, ^53^, ^55^, ^58^, ^63^, ^64^, ^67^, ^68^, ^70^, ^72^, ^73^, ^74^, ^75^, ^81^, ^82^, ^83^, ^84^, ^85^^,^
5 **Anexiety and depression**, it can cause functional deterioration and negatively impact the quality of life. It can also be related to suicidal thoughts, stress and attitudes, mentalities or thoughts of the patient that can negatively affect both health and the social and psychological environment, this factor was considered by. 51, 52, 55, 56, 58, 59, 64, 66, 68, 69, 72, 73, 75, 80, 85, 86, 87
6 **Severity of disease**, it is within the biological component of biopsychosocial factors. This variable is associated with problems generated by the disease and its progression. It generally refers to the level of impact that a condition or disease has on a person’s health status^88^ This factor was considered by.^7^^,^^29^^,^^39^^,^^46^^,^^50^, ^51^, ^56^, ^57^, ^60^, ^65^, ^66^, ^67^^,^^75^^,^^89^, ^90^, ^91^
7 **Ability/capacity to study or work**, which is used to identify those patients who have difficulties in continuing their studies or work due to their clinical condition. This factor was only considered by ^7^^,^^29^^,^^40^^,^^41^^,^^46^^,^^55^, ^65^^,^^66^^,^^75^^,^^79^^,^^80^^,^^92^, ^93^, ^94^, ^95^
8 **Time on waiting list**, it refers to the time that the patient has been on the waiting list (for example, days, weeks, months, or years). This is a variable that patients consider and emphasize when they have a health problem and was considered by.^29^^,^^46^^,^^47^, ^49^, ^50^, ^51^, ^57^, ^63^, ^66^, ^67^, ^71^, ^73^^,^^83^^,^^96^
9 **Age of patient**, it is a factor that, together with others, can determine the preoperative risk and, therefore, predict the risk of a bad result. The age of the patient was mentioned by.^44^^,^^45^^,^^49^, ^53^, ^57^, ^66^, ^67^, ^71^^,^^83^^,^^87^^,^^89^^,^^96^, ^97^, ^98^
10 La **Capacity to participate in social and family activities**, it indicates whether the patient on the waiting list has difficulties performing domestic and family activities^29^ and was considered by.^7^^,^^29^^,^^50^, ^55^, ^56^, ^64^, ^66^, ^67^^,^^75^^,^^79^^,^^94^^,^^95^
11 **Procedure effectiveness of the /expected improvement**, in elective surgery, it is crucial to determine both patient prioritization and surgical outcomes. By prioritizing patients who are expected to have the most significant improvement in quality of life and functional capacity, healthcare systems can optimize resource use and improve patient satisfaction.^99^ In our review, this factor was considered by.^7^^,^^29^^,^^39^^,^^46^^,^^50^, ^57^, ^60^, ^65^, ^67^^,^^82^^,^^100^^,^^101^
12 **Expected post procedure complications**, significantly affect clinical and economic outcomes, such as organ dysfunction, mortality, and hospital readmissions.^102^ In our review, this factor was considered by.^39^^,^^40^^,^^46^^,^^47^, ^48^, ^50^, ^60^, ^66^, ^67^^,^^81^^,^^83^
13 **Patient’s socioeconomic conditions/economic impact**, it is a variable that includes the patient’s socioeconomic level, whether he or she has high or low economic resources, whether he or she has lost income or independence, the patient’s ability to pay, and the economic impact are also associated. This variable is mentioned in the following articles.^40^^,^^42^^,^^45^, ^50^, ^59^, ^64^, ^67^, ^72^^,^^89^^,^^103^^,^^104^
14 **Issues with mobility or paralysis** are factors that are associated with problems in patient movement; this factor is one of the components of different tools such as the EuroQol-5D, Quality of life (QoL) (EQ-5D-3 L), and the Oxford Knee Score (OKS). The articles that use tools to measure this criterion found in our systematic review are^49^, ^51^, ^52^, ^55^, ^64^, ^68^, ^72^, ^73^, ^74^^,^^94^:
15 **Symptoms and their duration at the time of inclusion or while on the waiting list** are factors that correspond to symptoms at the time of inclusion or the deterioration and development of new symptoms during the waiting list. It also includes the importance and duration of the symptoms and, depending on the disease, the frequency with which these symptoms occur. The articles that address this topic are: .^39^, ^52^, ^54^, ^60^, ^62^, ^63^, ^64^^,^^100^^,^^105^^,^^106^
16 **Personal care** It is a variable that evaluates a person’s ability to perform basic personal care activities, such as washing, feeding, and performing personal hygiene tasks. The inability to perform these activities could negatively affect the patient’s health and, therefore, require some degree of prioritization. The articles in which this factor was included are the following: .^39^^,^^52^, ^55^, ^68^, ^72^, ^73^^,^^79^^,^^90^^,^^94^^,^^105^
17 **Impact on the disease if surgery is delayed**, this factor may be relevant depending on the type of elective surgery, since delaying surgery deteriorates and worsens the patient’s condition and the postoperative results. This factor was considered by.^41^^,^^44^^,^^45^^,^^46^^,^^48^^,^^57^^,^^60^^,^^61^^,^^107^
18 **Need for a caregive** It refers to whether the patient needs to be cared for due to his illness while he is on the waiting list, this implies that it affects the quality of life of another person and can be related to the severity of the illness. This factor was mentioned in.^7^^,^^29^^,^^39^^,^^46^^,^^65^^,^^67^^,^^93^
19 **COVID-19 vulnerability level**, it is related to the risk of exposure to COVID-19 from non-surgical treatment compared to surgery and also includes the patient’s exposure to COVID-19 and their attitude and behavior regarding whether they follow precautionary measures for COVID-19, as well as the degree of risk and family and personal history that may exist regarding this disease. This factor is addressed by.^40^^,^^44^^,^^45^^,^^53^, ^62^, ^68^^,^^100^^,^^103^
20 **Impact on the procedure if surgery is delayed** It can be decisive depending on the disease because, many times, the delay of elective surgery can cause the procedure to become more complex and a priority. The articles that considered this factor are the following: .^44^^,^^45^^,^^46^^,^^47^^,^^48^^,^^50^^,^^107^
21 **Surgical team composition and size** can help prioritize patients and reinforce the patient’s decision to undergo surgery, this factor was mentioned in.^40^^,^^44^^,^^45^^,^^47^^,^^48^^,^^97^
22 **Duration of benefit**, it is a factor used to prioritize the patient depending on the benefit duration once the surgery is completed. This factor helps maximize the limited resources available. The articles that addressed this factor are: .^39^^,^^41^^,^^82^^,^^95^^,^^101^^,^^106^^,^^108^
23 **Being a caretaker** It is a relevant social factor when undergoing surgery and defining the degree of prioritization of the patient since the existence of a person who depends on the patient who will undergo surgery may be relevant when prioritizing the surgery. Some authors who considered this variable are: .^7^^,^^29^^,^^46^^,^^55^, ^57^, ^67^^,^^79^
24 **Ethnocultural, ethnicity, ethnic-racial and cultural minority groups**, it is a factor to consider when prioritization policies favor certain social groups or when the disease affects explicitly certain ethnic groups, the articles that addressed this factor are.^64^^,^^66^^,^^87^^,^^92^^,^^97^^,^^104^^,^^109^
25 **Urgency of procedure** This variable indicates the level of urgency of each patient on the surgical waiting list. All of this is based on each patient’s clinical characteristics and psychosocial factors.^29^^,^^46^^,^^51^, ^67^^,^^96^^,^^97^
26 **Length of stay in hospital (LOS)**, it refers to the time that the patient remains in the hospital; this may be limited by the capacity of the hospital facility, the number of stretchers, and the availability of medical staff, among others. This factor is used in.^44^^,^^45^^,^^47^^,^^48^^,^^100^^,^^107^
27 **Alternative method (availability/effectiveness)**, it is a factor that responds to the availability and effectiveness of treatment other than surgery to treat the patient’s disease. The absence of alternative methods implies a higher priority for elective surgery.^42^^,^^44^^,^^48^^,^^93^^,^^100^^,^^107^
28 **Risk of death** in surgical procedures is generally high and depends mainly on the disease, types of procedures, and patients. This factor is associated with the likelihood or risk of a patient dying while waiting for the surgical procedure and is mentioned by.^40^^,^^46^^,^^57^^,^^83^^,^^106^^,^^108^
29 **Gender**, it is a factor that is generally considered by clinical criteria, where it can have a direct impact on the specific pathology or on the hormonal and symptomatic considerations of the patient; the articles that mentioned this variable are.^49^, ^67^^,^^87^^,^^89^^,^^92^^,^^104^
30 **Rurality / Distance / Difficulty in reaching a medical center**, it is a factor that, given the patient’s psychosocial condition, it evaluates whether he or she has difficulty moving to and from the hospital or medical center due to connectivity, long distances, or locomotion. This factor was considered by.^29^^,^^51^, ^64^, ^67^^,^^89^^,^^96^
31 **Duration of procedure**, it is a factor that considers the time that the surgical procedure lasts and is mentioned by.^44^^,^^45^^,^^46^^,^^48^^,^^63^ It is a relevant clinical criterion if other factors are considered, such as comorbidities, type of operation, and disease, since it could generate complications.
32 **Clinical factors**, it is a factor that allows to complement and inclusion of aspects not addressed in the other variables considered by the authors, such as the severity of the condition, medical urgency, and availability of beds, among others.^72^^,^^92^^,^^93^^,^^96^^,^^110^
33 **Extent of impairment in visual function**, it is a variable mainly present in ophthalmology and allows measuring the decrease or loss of the ability to perceive visual stimuli. The articles found that include this factor are: . ^,^^45^^,^ ^55^^,^ ^79^^,^ ^93^ ^94^
34 **Physical functioning, cognitive functioning** It covers the physical and mental skills necessary to process information, make decisions, remember, and perform activities. It involves the appropriate interaction of the musculoskeletal, cardiovascular, and respiratory systems. Articles that employed this factor are: .^67^^,^^69^^,^^80^^,^^83^^,^^86^
35 **Patient-derived impact on life**, it is a holistic factor that includes social interaction, personal interaction, the ability to fulfill responsibilities towards others, self-care, personal safety, and leisure activities. Articles that considered this factor are: .^60^^,^^101^^,^^106^^,^^109^^,^^110^

The factors by clinical area found in this systematic review are presented below.

### Otorhinolaryngology

For the clinical area of Otorhinolaryngology, we found two articles. The first one presents an ad-hoc clinical history form that captures the biopsychosocial condition of patients, followed by a dynamic scoring scheme that recognizes that patients’ conditions evolve differently while waiting for the required elective surgery and establishes a methodology for prioritizing and selecting patients based on the corresponding dynamic scores and additional clinical criteria (20 prioritization factors), obtaining as results a quantitative and qualitative improvement to the previous prioritization method of a high complexity hospital.^29^

In^7^ the authors established a network method that predicts the priority order of anonymous patients entering the SWL and also presents a dynamic quantification of the risk of waiting patients and a patient selection protocol based on a dynamic update of the SWL based on the prioritization components, risk, and clinical criteria.

Finally,^66^ focuses specifically on endoscopic sinus surgery and investigates patients’ preoperative concerns, identifying clinical factors such as anticipated post-procedure complications, anesthesia requirements, revision surgery, pain, and disease severity. Also identifies psychosocial factors such as time on the waiting list, the patient’s age, ability to work and/or study, ability to participate in social and/or family activities, the patient’s attitude, communication and ethnocultural factors.

**Fig. 4:**
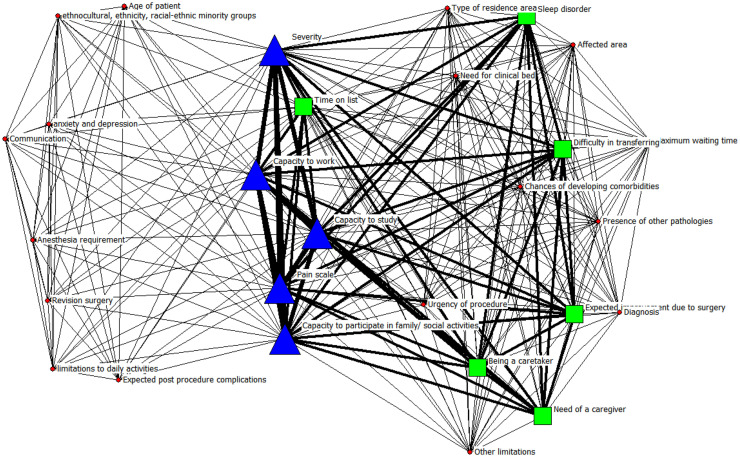
Otorhinolaryngology factors.

In Fig. 4, a triangle represents a frequency of 3; that is, the factor is considered in three articles, a square represents a frequency of 2, a circle represents a frequency of 1. It can also be observed that the thickness of these lines corresponds to stronger relationships between factors. We can observe that the factors that are present in the three articles are the severity, capacity to study, capacity to participate in family/social activities, pain, and capacity to work. The size of the nodes is proportional to the frequency with which the factors are mentioned in the articles.

### Urology

**Fig. 5:**
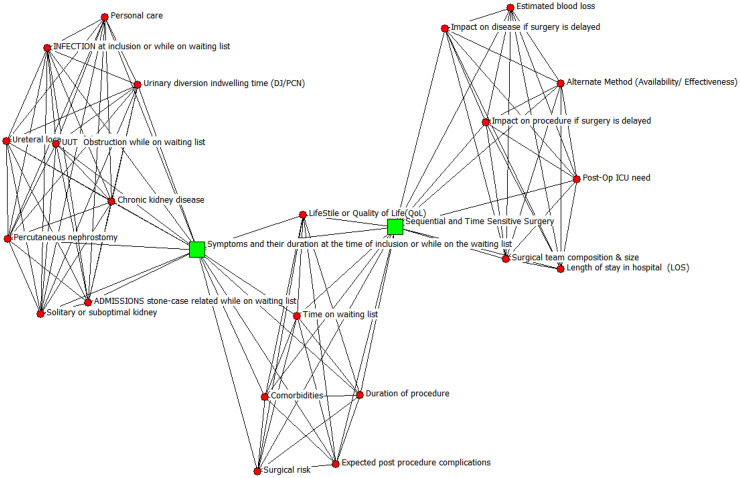
Urology factors.

In Fig. 5 is possible to identify that the factors, sequential and time sensitive surgery and symptoms and their duration at the time of inclusion or while on the waiting list, are those that appear most frequently,^111^ Analyzes the existence of inequality in access to care for elective surgery on socioeconomic indicators, while^100^ implemented a safe system for the provision of urgent elective surgical care at a COVID-19 clean site.

The size of the nodes is proportional to the frequency with which the factors are mentioned in the articles, as well as the figure; a circle represents a frequency of 1, a square a frequency of 2.

### Ophthalmology

In the area of ophthalmology, specifically cataract surgery, the following clinical factors were found: Preoperative visual acuity of the eye with cataracts, preoperative visual acuity of the contralateral eye, predicted visual acuity after surgery, glare, Fuchs’ dystrophy, hypertensive retinopathy, chronic simple glaucoma, corneal scar, age-related macular degeneration, other retinal diseases, retinal vascular disease, epithelial basement membrane disease, diabetic retinopathy, moderate or severe dry eye, epiretinal membrane disease, congenital/developmental conditions.

Other non-clinical (psychosocial) factors found are the degree of impairment in visual function, the ability to work, care for dependent persons or work independently, concerns about safety and injury, caring for one’s health, other significant disabilities, the ability to run local errands, the ability to manage household affairs and finances, the ability to assist others, the ability to participate in social and personal relationships and finally to participate in active recreational activities.

Other factors related to both clinical and biopsychosocial factors are night driving, the state of the contralateral eye, the complexity of the case, contrast sensitivity, mobility problems, or paralysis.

The Western Canada Waiting List Project (WCWLP) successfully developed and tested the validity of priority-setting criteria for cataract surgery.^94^ The WCWLP instrument includes both clinical and quality-of-life criteria for prioritization and has been used as a basis for the development of other instruments. The Swedish NIKE system,^93^ the New Zealand priority criteria,^109^ and the prioritization tool developed by^112^ were adapted from the WCWLP measurement instrument to fit the needs of their respective patients.^94^ modifies the WCWLP cataract prioritization criteria to emphasize Health-Related Quality of Life (HRQoL) (SVRS) and to examine critical aspects of reliability using a modified Delphi process.

In Khan et al.^71^ 7 factors are identified related to the elective corneal graft waiting list for Penetrating Keratoplasty (PK, full-thickness corneal graft) where patients requiring a corneal graft are assessed and scored by visual acuity in the graft eye, patient age, patient occupation, condition of the fellow eye, pain and discomfort experienced, risk of graft failure, and number of years the patient has been on the waiting list.

The eCAPS and Catquest-9SF questionnaires show some agreement with physician-reported appropriateness and patient-reported prioritization according to.^72^^,^^77^ In addition, the Catquest-9SF questionnaire was found to have excellent psychometric properties^78^ and, therefore, to be a valid measure for assessing a patient’s prioritization for cataract surgery.^79^ In contrast, eCAPS is not sensitive to differentiating patients referred for cataract surgery in Ontario, Canada, who had impaired visual functioning.^79^

The factors that are most frequently mentioned within the area of ophthalmology are limitations in daily activities, followed by the degree of deterioration in visual function, pain, the ability to work and/or study, personal care, quality of life, and a subgroup of variables that are related to daily activities (Satisfaction with vision/sight, reading text in a newspaper, recognizing the faces of people you know, looking at prices of purchased products, walking on uneven ground, doing sewing, crafts, reading subtitles on TV, trying to carry out a favorite hobby.^45^^,^^55^, ^71^^,^^76^, ^77^, ^78^, ^79^^,^^85^^,^^93^^,^^94^^,^^109^

### Cardiology

Through individualized surveys of specialists, general practitioners, nurses, patients, and their families^84^ identified and assessed a transparent and objective system for prioritizing patients on the waiting list for varicose vein surgery, which allows access to health services to be organized based on 5 factors: clinical manifestations (46.1 % of relative importance), varicose vein size (8.2 %), complications (18.3 %), influence on quality of life (18.2 %, measured with a Chronic Venous Insufficiency Quality of life Questionnaire [CIVIQ]) and aggravating work factors (9.2 %).

On the other hand^62^ evaluates a symptom-based Atrial Fibrillation (AF) ablation prioritization scheme for waitlist management compared with patient-completed quality of life (QoL) scores. Factors influencing patients’ quality of life were identified using the Atrial Fibrillation Effect on QualiTy of Life (AFEQT) and the EuroQol 5D (EQ5D-5 L) questionnaires, both of which are tools that help to identify patients at risk of admission beyond physician assessment. Regular exercise is associated with improved Quality of Life (QoL) in patients awaiting AF ablation, and patients with comorbidities have lower quality of life scores.

Doshmangir et al.^50^ develops a prioritization framework for patients requiring coronary artery angiography,^86^ attempts to establish agreement between patient and surgeon in assessing frailty, physical function, and social functioning. Finally, a longitudinal cohort study exploring the role of psychosocial factors was conducted by,^81^ concluding in a non-generalized manner that surgical complications affect patient physical and mental functioning.

The most frequently reported factors in these articles include anticipated post-procedure complications, comorbidities, and the patient’s quality of life.^50^^,^^62^^,^^81^^,^^84^ Other, no less important factors inherent to the disease or to the surgery are presented less frequently because they do not correspond to the same pathology. Examples of these factors are the size of the varicose vein,^84^ the number of myocardial infarctions (heart failure rate),^50^ and the frailty score.^86^

### Bariatric surgery

Public preferences in patient prioritization decisions for bariatric surgery are considered by^61^ using data from two Citizens’ Juries (CJs) and a Discrete Choice Experiment (DCE) considering 7 factors; demonstrated commitment to lifestyle change, very severe obesity (BMI >50 kg/m^2^), whether you have obesity-related comorbidity, severe obesity (BMI 40‒50 kg/m^2^), family history of obesity, additional month on waiting list and an additional % chance of maintaining substantial weight loss. The degree to which criteria were considered important was consistent across participants and the jury, there may be a small impact on the relative importance of criteria. CJs may clarify the underlying logic but may not provide substantially different prioritization recommendations compared to a DCE.

Other research supports several of the above factors in bariatric surgery,^82^ which developed a tool to help prioritize obese patients for bariatric surgery in New Zealand. In contrast,^101^ validates the National Bariatric Prioritization Tool (NBPT), developed in New Zealand (AoNZ) using real-world patient data.

The factors most frequently presented in these articles are surgical risk, the efficacy of the procedure, surgical benefit, and the likelihood of achieving maximum benefit with respect to diabetes control.^82^^,^^101^

#### Orthopedic surgery

Tebé et al.^65^ assesses the impact of implementing the Priority System in elective surgery (PKA) developed by (AQuAS.

The COVID-19 pandemic has influenced factors contributing to patients’ decisions for arthroplasty,^68^ highlights the importance of patient involvement to ensure optimized delivery of elective surgery by assessing factors using global Quality of Life (QoL) (EuroQol Five-Dimension, Three-Level [EQ-5D-3 L]) and joint-specific QoL (Oxford Hip or Knee Score), while^53^ concludes that Patients with worse hip function, as measured by the mHHS, are more willing to proceed with surgery in times of the (COVID-19) pandemic. On the other hand, clinical decision-making that sets inflexible cutoff points for BMI, HbA1c, and smoking status will worsen current racial, ethnic, gender, and socioeconomic disparities and limit access to surgery.^104^

In England, wait time prioritization policies that explicitly prioritize critically ill patients with a high marginal disutility of waiting,^49^ identified that those awaiting hip and knee replacement who experience more severe pain and immobility have shorter waits than those with less severe pain, and identified factors such as time on the waiting list, patient age, gender, comorbidities, and social security benefits as potentially impacting prioritization.

In the orthopedic area, quality of life and clinical frailty of patients waiting for hip or knee arthroplasty for more than six months were independently associated with the level of clinical frailty, which may be useful for prioritizing surgical waiting lists according to,^73^ on the other hand, the relationship between the preoperative Oxford score for hip and knee and the change in health-related quality of life after total hip and knee arthroplasty helps to inform rationing decisions.^74^ Furthermore, in two scenario-based experiments conducted by^87^ for both non-traumatic and trauma-related pathophysiologies, they noted that unhelpful thoughts, distress, and social problems were reasons to consider addressing mental and social health in treatment, relatively independently of pathophysiology.

#### Abdominal hernia surgery / abdominal wall hernia surgery

The use of a self-administered questionnaire to reduce waiting times in consultations of potential candidates for elective lumbar spine surgery is used by.^52^ On the other hand,^91^ evaluates the waiting lists for abdominal wall hernia repair (incisional vs. inguinal ventral hernia) by defining the following explicit prioritization criteria:(mFI-11): hernia complexity, patient frailty using the modified Frailty Index (mFI-11) and consumption of analgesics for the hernia, in addition the severity of the disease was measured using questionnaires (EQ-5D, COMI-hernia, HerQLes) of Health-Related Quality of Life (HRQoL).

The factors most frequently present in this clinical area are pain/discomfort, limitations to daily activities, the presence of comorbidities, anxiety and depression, mobility problems and paralysis, the patient’s Quality of Life (QoL), and the severity of the disease, among others.^40^, ^41^, ^42^^,^^49^, ^52^, ^53^, ^54^, ^56^, ^57^, ^65^, ^68^, ^69^, ^73^, ^74^^,^^87^^,^^91^^,^^104^^,^^106^

### General surgery

Many biopsychosocial factors found are not limited to the clinical area but can be extended to multiple clinical areas. An example of this can be found in^,^^39^^,^^46^^,^^47^^,^^48^ 51, 60, 67^,^^83^^,^^89^^,^^92^^,^^110^ where the factors used were not limited by the type of surgery or by the clinical area of the elective surgery.

**Fig. 6:**
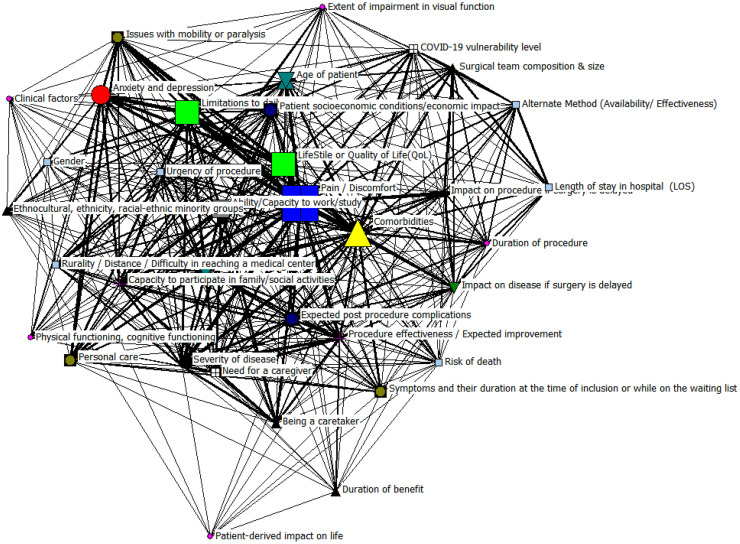
Cross-cutting factors.

The factors with the highest degree of centrality are the key points of this network. In this case, the factors are pain (209), comorbidities (150), limitations of daily activities (117), severity of the disease (114), quality of life (QoL) (107), time on the waiting list (105), effectiveness of the procedure (89) among others, this can be observed visually in the Fig. 6 where the size, color and shape of the node indicates the frequency of the factor.

The importance of a node in a network, determined by the centrality of the vector, taking into account both the number of connections that node has and the importance of the nodes to which it is connected, as well as the degree centrality, the first 6 factors maintain their order of importance. The seventh factor with the highest vector centrality is anxiety and depression, with a value of 0.22, followed by the ability to participate in family and social activities (0.20).

Below, we detail the studies carried out in other surgical sections.

#### Rheumatology

Critical factors identified by^95^ about rheumatology services were the effect of the problem on the patient’s life and role, the presence of inflammatory rheumatic disease, the appropriateness of current treatment, and the ability of the rheumatologist to influence current symptoms and future prognoses.

#### Plastic surgery

The prioritization of plastic surgery by^75^ identifies biopsychosocial factors in cases where changing the underlying anatomy allows patients to feel better and integrate into society rather than restore physical function. He concludes that a health prioritization process centered on the patient and their informed preferences could be implemented considering the factors and variants in each patient that affect their Quality Of Life (QoL), such as tastes, predominant individual and social values, economic situations, lack of access to education, autonomy, personal relationships, knowledge, and physical coercion.

#### Maternal-fetal surgery and in vitro fertilization

^108^ assesses physicians’ ratings of the importance of 9 considerations relevant to maternal-fetal surgery using a discrete choice experiment contained in a national survey of neonatologists, pediatric surgeons, and maternal-fetal medicine physicians.^108^ Assesses the importance of 9 considerations relevant to maternal-fetal surgery: (1) Neonatal benefits: the child benefits from the operation; (2) Maternal autonomy: the pregnant woman’s right to make decisions; (3) Risk of prematurity: increased likelihood that the child will be born preterm; (4) Risk of stillbirth: increased likelihood that the fetus will die or the baby will die shortly after birth; (5) Risk of maternal complications: increased likelihood that the woman’s health will be affected; (6) Risk to future reproductive health, such as risk of uterine rupture with future pregnancies requiring cesarean deliveries; (7) Maternal social support: ensuring that the pregnant woman has physical and emotional support; (8) Impact on other family members: the impact on parents, siblings or grandparents; and (9) Maternal psychological benefit: increased maternal satisfaction or self-esteem.

On the other hand, In Vitro Fertilization (IVF) is an assisted reproduction technique that involves a minor surgery called follicular aspiration to extract eggs from the woman’s body where^98^ looks at how fertility clinics prioritize patients for IVF cycles with limited funding in Ontario. Through a survey, he determined the factors used by clinics to prioritize patients where the majority use the first come, first-serve basis (90.9 %), however, there are other clinics that also consider older patients (81.8 %), patients about to lose their eligibility for funding (63.6 %); duration of infertility (36.3 %); and duration at the current clinic (36.3 %).

#### Endoscopic sinus surgery

Endoscopic Sinus Surgery (ESS) is an elective procedure for chronic rhinosinusitis that carries a clear and defined set of risks,^66^ identifies the concerns of patients before undergoing elective sinus surgery and emphasizes the importance of a patient-centered care approach. Clinical factors, such as anticipated post-procedure complications, anesthesia requirement, revision surgery, pain, and disease severity, are identified. In addition, psychosocial factors such as time on the waiting list, patient age, ability to work and/or study, ability to participate in social and/or family activities, patient attitude, communication, and ethnocultural factors are identified.

#### Elective surgeries in gynecology

^90^examines the various aspects of prioritizing non-emergency gynecological care, including outpatient appointments and elective surgery, how innovative pathways have evolved in response to need, what some of the barriers to implementing them have been, and how this has impacted individual gynecological specialties more broadly, identifying factors related to disease severity, patient willingness to undergo surgery, patient preferences and expectations, and communication.

#### Liver transplant

In patients with cirrhosis, frailty represents a state of global physical dysfunction associated with a multiplicity of factors, including muscle wasting, malnutrition and undernutrition, and functional impairment, which is an independent predictor of adverse surgical outcomes. Therefore, its incorporation into clinical practice could result in better clinical decision-making.^85^

## Discussion

In the literature, we find various biopsychosocial factors that are used, analyzed, and observed in the prioritization of patients on the elective surgical waiting list, such as pain, the ability to participate in social and family activities, being a caregiver, and being cared for, among others.

The selection of appropriate tools to measure the factors depends on the clinical area and the variables to be considered. The methods used in these studies are mainly through self-administered questionnaires, the Delphi method, scoring systems (MeNST) and its factors (disease, procedure, and patient factors), health questionnaires such as (Oxford Knee Score, EQ-5D-5 L, EQ-5D-3 L, PEG, mF1–11, PGQ-9, CIMD), whose descriptive system allows the identification of several dimensions of health.

The appropriate combination of tools helps to establish relationships between biological, physical, and social factors,^56^ identifies psychological and electrodiagnostic factors associated with limb disability in patients with carpal tunnel syndrome using tools such as QuickDASH, this questionnaire consists of 11 items from the original 30-item DASH that addressed upper extremity symptoms and disabilities, the HADS designed to detect and quantify Depression (HADS-D) and Anxiety (HADS-A), The PCS a 13-item instrument with three subscales assessing rumination, magnification, and helplessness to assess the mental status of patients with pain, and the EMG-NCS which consists of a set of tests performed to assess the health of the body’s nerves and muscles.

In the literature, we find four groups of factors that are widely analyzed in the literature: factors related to the disease, factors related to the patient, factors related to time, factors related to the surgical procedure, and psychosocial factors.^46^

Consideration of biopsychosocial factors in the prioritization of patients on the elective surgical waiting list is increasingly important in decision support models,^28^^,^^29^^,^^46^ and several studies found in this review support this claim. In addition, some articles investigate patient-centered prioritization, where the patient’s opinion is weighted and considered to improve different clinical aspects, such as reducing elective surgical waiting lists, reducing waiting times, and mitigating the impact on patients. Other studies focused on factors that impact patients.^83^

These results obtained concerning the biopsychosocial factors of elective surgical waiting lists are based on the fact that the concepts and limits of health and illness are in themselves biopsychosocial; on the other hand, the opinion of the doctor and the biological factors that he may consider, both of the disease and the patient, must not be neglected and set aside. Furthermore, the biopsychosocial concept is included in various reviewed studies.^28^^,^^29^^,^^46^ This holistic approach is not used in other studies not considered in this review that address elective surgical waiting lists from a perspective focused on supply and demand or hospital management, where psychosocial aspects related to the patient are not considered.^113^^,^^114^

For priority allocation to be successful and effective, outcomes that accurately predict benefits must be identified, one challenge being that standard outcome metrics are not always aligned with the critical benefits expected by patients.^76^

## Conclusion

Long waiting times, inequalities, and lack of prioritization in access to health services are major challenges faced by both public and private health systems. The development and validation processes of Patient Prioritization Tools (PPT) in non-urgent healthcare settings, mainly in the context of elective surgeries, could help manage access to care in an equitable manner, as well as decision-making support systems.

In this article, we conducted an exhaustive review of the literature following a PICOC methodology, allowing us to analyze 71 articles relevant to the topic under study. In the analysis, we identified 35 transversal biopsychosocial factors, of which the most frequently found in the literature were pain (which has a biological component), comorbidities (which have a biological component), limitations to daily activities (which have a social component), and lifestyle (quality of life) (which has psychological, social, and biological components). Also, by clinical area, we identified the most frequent factors and their relationships with other factors, making them more relevant to consider in the prioritization of patients. This allowed us to establish the factors and how they are related by clinical area. For example, in otorhinolaryngology, severity, capacity to work, pain, capacity to study, and capacity to participate in family/social activities are key factors highly connected to other factors.

We identified biopsychosocial factors that can be used as input for policymakers, researchers, and health workers to conduct and support projects to assess the facilitators and barriers to implementing such innovations. Further research is also needed to explore the outcomes of using PPTs and their effects on waiting times in clinical settings.

Although we were able to identify recommended biopsychosocial factors to consider in the development of reliable and valid PPTs for non-urgent services, we believe that facilitators and barriers to implementing such systems should be assessed. Further research is also needed to explore the outcomes of using PPTs that consider biopsychosocial factors, as well as their effects on waiting times in clinical settings.

Some limitations of this study to consider are that the clinical factor related to the medical diagnosis and the disease depends on variables such as the type of disease, symptoms, environment, and availability of medical equipment, among other variables. This can cause the importance of a medical factor not to be reflected in the results because they are not grouped.

## Abbreviation

NBPT, The National Bariatric Prioritization Tool; AoNZ, Aotearoa New Zealand; PKA, Primary Knee Arthroplasty; AQuAS, The Agency for Health Quality and Assessment of Catalonia; MeNST, Medically Necessary Time-Sensitive score; EQ-5D(3 L), Instrument measuring health status. Five items measure the participant’s current general health in domains of mobility, self-care, usual activities, pain/discomfort, and anxiety/depression^64^; PEG, Pain, Enjoyment of Life and General Activity scale, used to measure participant’s level of pain^64^; PHQ-9, Patient Health Questionnaire used to assess participants’ symptoms and functional impairment^64^; CIMD, Canadian Index of Multiple Deprivation, measures four domains of deprivation and marginalization at the neighborhood-level^64^;QuickDASH Quick Disabilities of the Arm, Shoulder, and Hand^56^; HADS, Hospital Anxiety and Depression Scale^56^; PCS, Pain Catastrophizing Scale^56^; EMG-NCS, Electromyography-Nerve Conduction Study.^56^

## Ethical considerations

In this research, we used secondary data from articles published. We follow methodologies widely used in literature reviews, such as PRISMA, PICOC, and others. Since our research does not constitute a clinical trial, observational study, diagnostic or prognostic study, or animal research, the CONSORT, STROBE, STARD, and ARRIVE guidelines do not apply. Also, because this study is a literature review, the intervention of the Ethics Committee, informed consent, and patients' details are not required.

## Authors' contributions

Fabián Silva-Aravena: Conceptualization; methodology; validation; investigation; data curation; writing-original draft; writing-reviewing & editing.

Jenny Morales: Methodology; validation; investigation; supervision; writing-original draft; writing-reviewing & editing.

Leonidas López Canales: Data curation; visualization; investigation; software; writing-reviewing & editing.

## Funding

This research was funded by the“ANID Fondecyt Iniciación a la Investigación 2024 n° 11240214”.

## Data availability

The datasets generated and/or analyzed during the current study are available from the corresponding author upon reasonable request.

## Declaration of competing interest

The authors declare no conflicts of interest.
